# Biocontrol of Carp: More Than Just a Herpesvirus

**DOI:** 10.3389/fmicb.2018.02288

**Published:** 2018-09-26

**Authors:** Kenneth A. McColl, Agus Sunarto, Matthew J. Neave

**Affiliations:** CSIRO-Australian Animal Health Laboratory, Geelong, VIC, Australia

**Keywords:** common carp, *Cyprinus carpio*, viral biocontrol, CyHV-3, specificity, efficacy, latency

## Viral biocontrol of invasive vertebrate pests

Viral biocontrol has twice been used successfully in Australia to control, but not eradicate, an important terrestrial pest species, the rabbit (*Oryctolagus cuniculus*). Lessons from those attempts (McColl et al., 2014) have been used to develop a viral biocontrol program for common carp (*Cyprinus carpio*), a cyprinid teleost fish that is the major vertebrate pest in Australian inland waterways (Koehn, 2004).

Cyprinid herpesvirus 3 (CyHV-3; Hedrick et al., 2000) was recognized as a potential biocontrol agent for carp (McColl et al., 2014), and it is now a central element of the National Carp Control Plan in Australia (http://www.carp.gov.au/). However, for most of the world, common carp are an important food source, and among the most farmed fish globally (Ronen et al., 2003). In carp aquaculture, CyHV-3 can be a devastating pathogen (Sunarto et al., 2005), and therefore, unlike Australia, the emphasis throughout much of the world is on viral control rather than carp control.

It is perhaps because of this dichotomy that numerous misconceptions and misunderstandings have arisen about CyHV-3 itself, and about the viral biocontrol program for carp in Australia. Here we present our view on each of these problematic issues.

## Misconceptions and misunderstandings

### CyHV-3 can infect fish, and other animals, apart from carp

The susceptibility of at least 22 species of fish to infection with CyHV-3 has been tested (summarized in Boutier et al., 2015). While none showed clinical signs of disease, viral DNA was detected by polymerase chain reaction (PCR) tests in at least 10 species, although always in small numbers of each species tested, and, when present, always in low copy numbers (Fabian et al., 2013). Similar results were found for plankton, freshwater mussels and some crustaceans (Kielpinski et al., 2010; Minamoto et al., 2011). These findings led to the view that many species of fish, and some invertebrates, could be infected, but not affected, by CyHV-3. However, none of these early studies used reverse transcriptase-PCR (RT-PCR) to prove that viral replication (the presence of viral mRNA) occurred in non-carp species (known as non-target species, NTS, in the context of viral biocontrol). An attempt by El-Matbouli and Soliman (2011) failed for technical reasons (Yuasa et al., 2012).

McColl et al. (2016b) not only tested the susceptibility of a range of animals to the virus, but they also used high doses of virus and conditions that favored virus infection. Apart from clinical and pathological examinations of NTS, tissues from NTS were also screened for viral DNA by PCR, and any DNA-positive samples were then examined by an RT-PCR (Yuasa et al., 2012) for viral mRNA. Of 1,355 samples screened for viral DNA, 109 were weak false-positives because all were negative for the presence of viral mRNA.

In summary, there was no evidence for any NTS being infected, let alone affected, by CyHV-3. These results are supported by the absence of substantiated disease reports due to CyHV-3 in any other species throughout the world.

### Mutations in CyHV-3 DNA, or recombination with other viruses, could change the host-range of subsequent viruses

While the specificity of the laboratory strain of CyHV-3 seems clear, further questions arise about the potential for genetic changes to occur in the field following release of the virus, and whether these changes could alter the host-range of the original virus. Lessons from field observations on the two rabbit biocontrol viruses in Australia, the myxoma virus (MYXV) and rabbit hemorrhagic disease virus (RHDV), partly address these questions (McColl et al., 2014). Despite mutations being recognized in the field for both MYXV (present in Australia for over 60 years; Kerr et al., 2015) and RHDV (present for over 20 years; Mahar et al., 2016), there have been no reports of either virus crossing species barriers in Australia.

Evolutionary studies (Geoghegan and Holmes, 2017) have suggested that it can never be certain that a biocontrol virus will not cross a species barrier. However, it was also demonstrated that, while host-jumping may occur with any DNA virus (such as CyHV-3), these jumps are invariably less frequent than for RNA viruses, and, when they do occur, the jump is invariably into a taxonomically closely-related species (Geoghegan et al., 2017). It is relevant that, firstly, there are no native cyprinids in Australia, and, secondly, that two species of native catfish, the most closely related native fish to carp in Australia, showed no evidence of virus replication when challenged with CyHV-3 (McColl et al., 2016b). Geoghegan et al. (2017) also noted that, for herpesviruses, host-jumping occurs on time-scales of millions of years, not the decades that will be required to control carp in Australia if CyHV-3 is complemented with another control measure (McColl et al., 2016a).

Recently, Gao et al. (2018) reported the first evidence for recombination in CyHV-3. In Australia, a virome study is underway to identify endemic viruses of carp, and those that could potentially recombine with CyHV-3. However, there are no compelling field observations overseas to suggest that genetic changes, of any sort, in CyHV-3 are likely to alter its host specificity.

### CyHV-3, alone, will control carp in australia

A critical lesson from viral biocontrol of rabbits in Australia has been that, if a biocontrol virus is to be successful, it must be complemented by another broad-scale control measure (McColl et al., 2014). This has been a step-wise learning process. While the use of MYXV revealed that a virus, alone, will not be completely effective, the use of RHDV then demonstrated that, although long-standing regional controls for rabbits (for example, poisoning, destroying rabbit warrens, trapping) could prolong viral efficacy, such methods were still insufficient for sustained control (Saunders et al., 2010).

From the outset, we have promoted the use of a genetic strategy(ies) or the next generation(s) of virus as essential complementary broad-scale controls for CyHV-3 (McColl et al., 2014, 2016a). Our critics, however, have chosen to ignore our commitment to these additional measures (Lighten and van Oosterhout, 2017; Marshall et al., 2018). Each of the currently available genetic strategies has deficiencies, either because they would involve genetically-modified fish (Thresher et al., 2014; Akbari et al., 2015), or require the expensive regular addition of modified fish to waterways (Cotton and Wedekind, 2007; Thresher et al., 2014). However, a recent new genetic approach (Maselko et al., 2017), although not an ideal complement to CyHV-3, provided optimism that further genetic advances will likely fill the void.

### Latency has been proven for CyHV-3

The ability to induce latent infections in their host is one of the hallmarks of herpesviruses (Stevens, 1994). While there is little doubt that CyHV-3 will eventually be shown to possess this characteristic, there is currently no definitive evidence to support this view. The case for latency for CyHV-3 has been built on observations of carp collected from the field, usually with a sparse clinical history, and no information on the dose of virus or the route of infection (Eide, K. E. et al., 2011; Eide, K. et al., 2011; Xu et al., 2013; Zheng et al., 2017).

Importantly, in many cases, wild-caught carp were collected from waters that were at non-permissive temperatures for CyHV-3, and then maintained at those non-permissive temperatures for experimental work (Eide, K. E. et al., 2011; Eide, K. et al., 2011; Reed et al., 2015, 2017; Lin et al., 2017). Herpesvirus latency occurs naturally at permissive temperatures for the virus, so *in vivo* studies on latency must also be conducted at permissive temperatures. From reported studies on CyHV-3, it is impossible to differentiate a latent infection from a low temperature-induced, low-level persistent infection with suppressed virus expression. The observation of almost a thousand-fold higher frequency of latently-infected cells in an experimental koi carp model compared with a recognized Epstein-Barr model of latency (Reed et al., 2015) is consistent with this view. Recognition of this difference will likely be important in understanding the epidemiology of the disease in carp (and therefore in developing approaches to viral biocontrol), but perhaps more importantly, for biosecurity (where molecular detection of latently-infected carp would prove much more difficult than those that were persistently-infected).

### Antibody responses to CyHV-3 may be detected in surviving carp for many months post infection

If CyHV-3 were to be released in Australia, then detection of specific antibodies against the virus in surviving carp will be important in monitoring the spread of the virus in wild carp populations. Interpretation of antibody data would be based on earlier studies that have suggested that specific antibodies can be detected for up to 1 to 2 years post infection (Adkison et al., 2005; Cabon et al., 2017).

However, Cabon et al. (2017) recognized that serial bleeding of surviving CyHV-3-infected carp would likely trigger viral reactivation with a subsequent boost to the titre of specific antibodies. Examination of the four studies that studied the dynamics of the specific antibody response to CyHV-3 (Ronen et al., 2003; Perelberg et al., 2008; St-Hilaire et al., 2009; Cabon et al., 2017) suggests that in each case reactivation of a latent or persistent infection was likely to have occurred. The results of such studies would then encourage the view that surviving carp remain seropositive for much longer than actually occurs in a natural setting.

It has long been recognized that, in sero-surveillance of fish, the serological status of fish populations is more consistent than individuals (Neave et al., 2017). Therefore, ideally, a serological test should be sensitive enough to assess the antibody status of individual fish, and, subsequently, allow correlation of this status with sensitivity to re-infection. Neave et al. (2017) have taken initial steps to achieve these aims.

### Indirect transmission of CyHV-3 between fish is as important as direct transmission

Although there have been no specific studies on the relative importance of direct versus indirect transmission of CyHV-3 between carp, observations on the biology of both the virus and carp have suggested two hypotheses (McColl et al., 2016a).

Firstly, it is likely that direct skin-to-skin contact is, by far, the most efficient form of transmission of virus, the implication being that indirect transmission by waterborne virus (following, for example, excretion from carp, or from birds or humans dropping dead, infected carp in virus-free locations) is likely to be much less efficient in transmission of CyHV-3. Secondly, we hypothesize that transmissibility will be favored by the evolution of low virulence strains of CyHV-3 that allow survival of many infected carp, with subsequent regular recrudescence of infection in, and direct transmission from, these survivors during breeding aggregations (McColl et al., 2016a). If correct, the implication of the latter hypothesis supports our earlier contention that virus, alone, is unlikely to be effective as a biocontrol agent for carp.

### There may already be viruses in australian carp that could potentially cross-react with CyHV-3, making biocontrol ineffective

Another important lesson from work on rabbit biocontrol with RHDV was that an unrecognized, cross-reactive and avirulent virus in the targeted pest species may confer protection from a biocontrol virus (McColl et al., 2014). Gao et al. (2018) have suggested that, based on genetic analysis of recognized lineages of CyHV-3, a variant(s) of CyHV-3 has been present in common carp for tens of thousands of years. This seems consistent with: molecular evidence for a CyHV-3-like virus in European common carp (Engelsma et al., 2013); an unusual variant in New York State (Grimmett et al., 2006); and, a possible variant, detected by PCR, in 13 of 14 wild common carp at a location in Oregon where no major CyHV-3 outbreaks have been documented (Xu et al., 2013).

Given the range in the severity of CyHV-3 outbreaks around the world in recent decades (Hedrick et al., 2000; Matsui et al., 2008; Thresher et al., 2018), it may become important to correlate, if possible, the nature and titre of variants in carp around the world with the likelihood of CyHV-3 outbreaks. In particular, if CyHV-3 is to be used in Australia as a biocontrol agent, it is essential that Australian carp be screened for avirulent variants of CyHV-3. A preliminary cyprinid herpesvirus PCR survey (based on a highly-conserved polymerase gene) of 849 carp in the Murray-Darling Basin failed to detect any cyprinid herpesviruses (McColl and St Crane, 2013); this finding will be compared with the current virome study.

## Final comments

We have always been aware that the release of an exotic virus into Australia's waterways, if it were to occur, would be a contentious event. Our aim from the outset has been to find reasons why release of CyHV-3 would *not* be a good idea. Consistent with this approach, we have already addressed some of the misconceptions and misunderstandings discussed in this review. However, all of these issues may become important as we continue to address the safety and efficacy of CyHV-3 as a potential biocontrol agent in Australia.

By openly addressing these issues, we hope to curtail ill-informed discussion about the virus without ever discouraging specific, evidence-based criticisms of the proposed use of CyHV-3.

## Author contributions

All authors listed have made a substantial, direct and intellectual contribution to the work, and approved it for publication.

### Conflict of interest statement

The authors declare that the research was conducted in the absence of any commercial or financial relationships that could be construed as a potential conflict of interest.
